# Fluorimetric Detection of Zn^2+^, Mg^2+^, and Fe^2+^ with 3-Hydroxy-4-Pyridylisoquinoline as Fluorescent Probe

**DOI:** 10.1007/s10895-020-02666-0

**Published:** 2020-12-19

**Authors:** Gabriel E. Gomez Pinheiro, Heiko Ihmels

**Affiliations:** Department of Chemistry and Biology, and Center of Micro- and Nanochemistry and Engineering (Cμ), University of Siegen, Adolf-Reichwein-Str. 2, 57068 Siegen, Germany

**Keywords:** Isoquinoline, Metal ion complexes, Fluorescent probe, Chemosensor

## Abstract

The suitability of 3-hydroxy-4-pyridylisoquinoline to operate as fluorescent chemosensor for the detection of metal ions was investigated. For that purpose, the interactions of the title compound with selected metal ions were investigated by absorption and emission spectroscopy. The complexation of Zn^2+^, Fe^2+^, Mg^2+^ with 1:1 and 2:1 stoichiometry leads to characteristic optical responses that depend significantly on the employed solvents, thus allowing for the fluorimetric identification and detection of particular metal cations in a matrix-based pattern analysis or by fluorimetric titrations.

**Supplementary Information:**

The online version contains supplementary material available at 10.1007/s10895-020-02666-0.

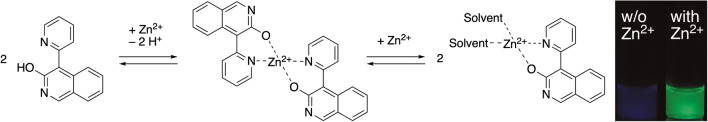

## Introduction

**Scheme 1:**
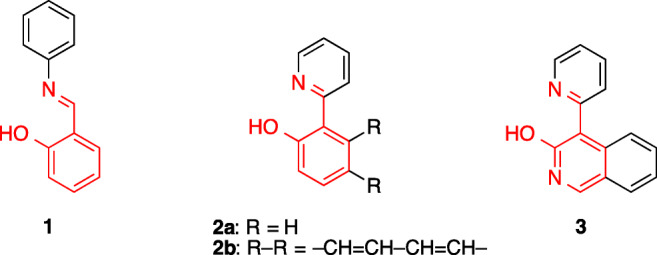
Structures of chemosensors **1** and **2** used the for the detection of metal ions and the structurally resembling 4-pyridyl-3-hydroxyisoquinoline (**3**).

The concentration of metal cations in biological media or in the environment is an essential parameter that determines – among others – the vital function of a bio- or ecosystem [1–4]. Thus, anomalous concentrations of particular cations may lead to harmful effects such as serious diseases as well as contamination and pollution. As a result, the development of sensitive and selective optical probes, also named chemosensors, for cation detection has attracted much attention in recent years [5–10]. Specifically, the development of sensitive and easy-to-use fluorimetric probes for biologically abundant metal cations, such as Zn^2+^ and Fe^2+^, has a special importance as imbalances in their levels may lead to serious health problems [11–19]. To this end, numerous fluorescent ligands, whose emission changes signficantly on cation complexation, have been developed and employed for the detection of metal ions in biological and environmental samples [5–10]. For example, aromatic 2-hydroxy-substituted Schiff bases (**1**) [11, 13, 18, 20–35] and hydroxyphenyl and hydroxynaphthylpyridines (**2**, Scheme 1) [17, 36–38] have been shown to act as colorimetric, fluorimetic and electrochemical probes for metal ions, because they form complexes with particular cations with high selectivity, which in turn leads to a significant change of their optical or electrochemical properties.

In this context, we have recently observed that the absorption and emission properties of the structurally resembling 4-pyridyl-3-hydroxyisoquinoline (**3**) and its derivatives are highly dependent on their environment in solution [39], and others have assessed these properties theoretically [40]. Namely, the changes in solvent polarity and pH affect the tautomeric equilibrium of **3** which in turn leads to changes of the emission color and intensity. Considering the fact, that the 4-pyridyl-3-hydroxyisoquinoline (**3**) has the same 2-(2-hydroxyaryl)pyridine unit as the established ligands **1**, **2a** and **2b** (Scheme 1), we reasoned that the former may also operate as an efficient ligand in metal-ion complexes. And based on our previous findings with **3** [39] we proposed that this complex formation is accompanied by a distinct change of its emission properties, so that it may be used as fluorescent probe for metal cations. To check this hypothesis we assessed the complexing ability of **3** toward selected metal ions by means of absorption and emission spectroscopy, and we investigated the effect of the complex formation on the emission properties. Herein, we will show that the emission color and intensity of compound **3** depend on the complexed metal ion and on the employed solvent, leading to characteristic fluorescence patterns in a series of solvents that enables the fluorimetric detection of metal cations by pattern analysis or by fluorimetric titrations.

## Results

### Photometric Titrations

**Fig. 1:**
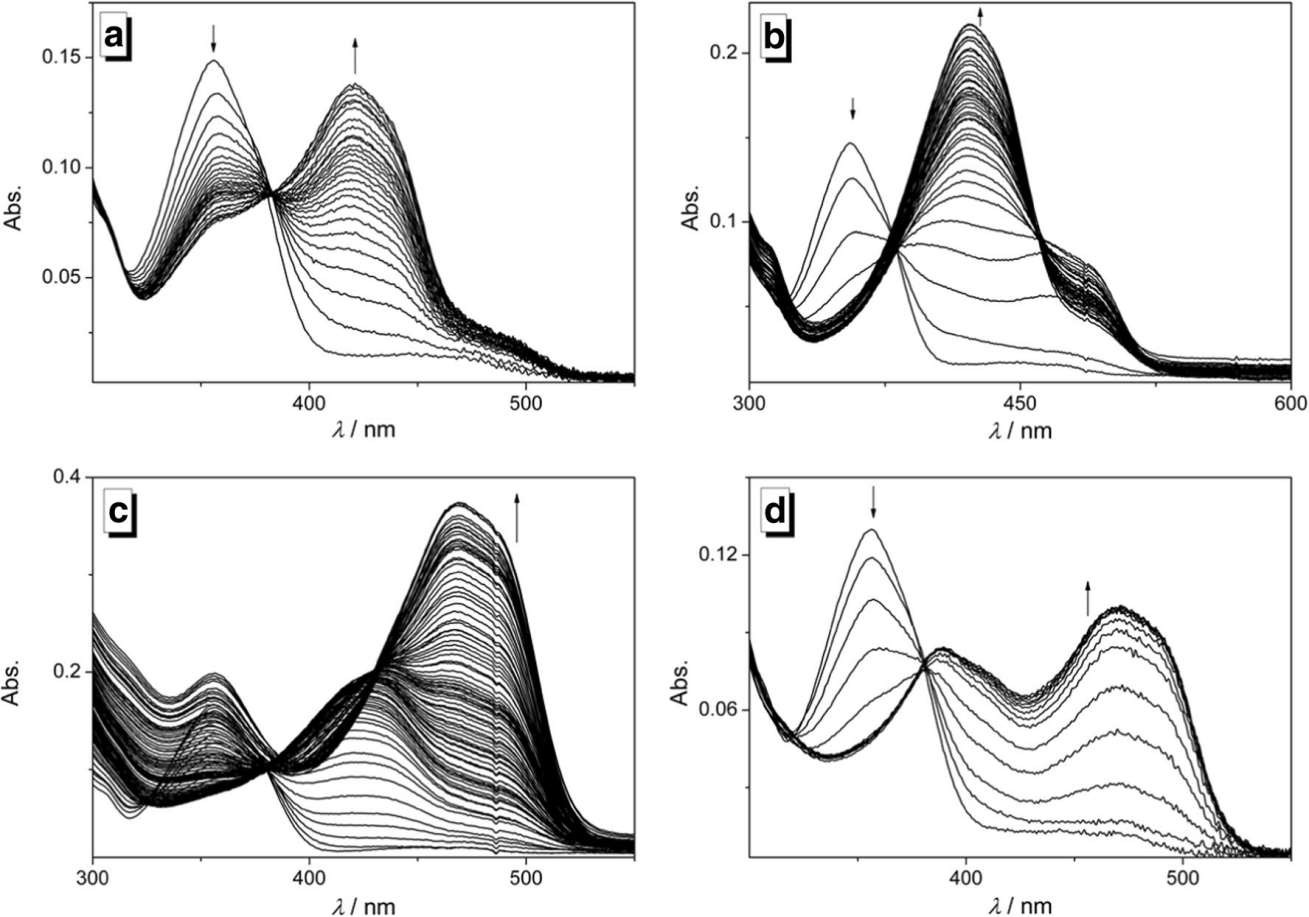
Photometric titrations of 3-hydroxy-4-pyridylisoquinoline (**3**) (*c* = 50.0 μM) with Mg^2+^ (**a**), Zn^2+^ (**b**), Fe^2+^ (**c**), and Pb^2+^ (**d**) in acetonitrile. The arrows indicate the development of absorption bands during titration

The formation of complexes between 3-hydroxy-4-pyridylisoquinoline (**3**) and representative metal ions Mg^2+^, Zn^2+^, Fe^2+^ and Pb^2+^ in acetonitrile was followed by photometric titrations. In each case, the addition of the metal ion to compound **3** caused a decrease of the absorption band of the ligand and the formation of new red-shifted bands. Notably, the development of new bands during titration was significantly different with the respective metal ions. Thus, the addition of Mg^2+^ and Zn^2+^ to ligand **3** led to the formation of a red-shifted band at 421 nm, along with shoulders at 492 nm and 485 nm, respectively. However, only in the case of Zn^2+^ the intensity of the red-shifted shoulder decreased during titration while the intensity of the maximum at 421 nm increased further (Fig. S1A). During the addition of Pb^2+^ two red-shifted absorption maxima of similar intensity developed steadily at 389 nm and 470 nm, whereas the addition of Fe^2+^ caused the initial development of only one new band at 424 nm, which was followed by the formation of two additional bands at 355 nm and 470 nm at higher metal ion concentrations (Fig. S1B). Only on addition of Mg^2+^ and Pb^2+^ to **3** an isosbestic point was maintained at 382 nm and at 381 nm, respectively, during most phases of the titration (Fig. 1), which may be explained by one dominant equilibrium over an extended range of metal ion-ligand ratio and closely resembling absorption spectra of the ligand in the 1:1 and 2:1 complexes.

The data from the photometric titrations of **3** with Mg^2+^ and Fe^2+^ in acetonitrile was used for the determination of the respective binding constants *K*_*b*_ and the ligand-metal ratio of the complexes (Table 1). Hence, at low concentrations (*c*_M_/*c*_L_ < 1) Mg^2+^ and Fe^2+^ form predominantly 2:1 complexes with the ligand, whereas with increasing metal-to-ligand ratio the 1:1 complexes are formed to more extent until both species are in equilibrium at the end of the titration (Fig. S2 and S3). Unfortunately, the determination of the stability constants of the complex of ligand **3** with Zn^2+^ and Pb^2+^ in acetonitrile was not possible from these data, presumably due the relatively large binding constant, as indicated by the significant changes of the absorption spectra even at very low metal ion-to-ligand ratios. Based on these results, the isosbestic points formed at some particular stages of the titration with Mg^2+^ and Pb^2+^ (Fig. 1a and d) may be explained by one dominant equilibrium over an extended range of metal ion-ligand ratio and closely resembling absorption spectra of the ligand in the 1:1 and 2:1 complexes in these cases.

**Table 1:** Photometric data and stability constants obtained from the titration of **3** with Mg^2+^, Zn^2+^, Fe^2+^, and Pb^2+^ in acetonitrile solution

	λ_abs_ / nm^a^	*K*_11_ × 10^4^ / M^−1 b, c^	*K*_21_ × 10^4^ / M^−2 b, d^	λ_fl_ / nm^e^	*Φ*_fl_^j^
Mg^2+^	421	3.6 ± 0.2	15 ± 1	497^f^	0.30
Zn^2+^	421	–	–	496^g^	0.35
Fe^2+^	424	3.9 ± 0.4	27 ± 3	538^h^	0.05
Pb^2+^	470	–	–	530^i^	0.02

^a^Long-wavelength absorption maximum. ^b^ Determined at 20.0 ± 0.1 °C from photometric titrations. ^c^ Binding constant of 1:1 complex. ^d^ Binding constant of 2:1 complex. ^e^ Red-shifted emission maximum. ^f^ λ_ex_ = 383 nm. ^g^ λ_ex_ = 380 nm. ^h^ λ_ex_ = 381 nm. ^i^ λ_ex_ = 379 nm. ^j^ Fluorescence quantum yield relative to coumarin 153 (*Φ*_fl_ = 0.38 in EtOH) [Ref. 41], λ_ex_ = 380 nm, estimated error: ±10% of the given values

**Scheme 2:**
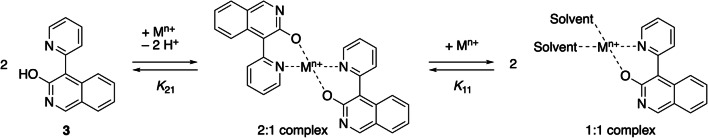
Complexation equilibrium of 3-hydroxy-4-pyridylisoquinoline (**3**) with metal ions.

All experimental data point to a complexation of the metal ions to 3-hydroxy-4-pyridylisoquinoline (**3**), which results in significantly different optical responses that clearly depend on the nature of the metal ion. Specifically, the photometric titrations showed that Mg^2+^ and Fe^2+^ form a 2:1 complex with ligand **3** at higher ligand-metal ratios whereas 1:1 complexes are formed with increasing metal-ion content (Scheme 2). The formation of 2:1 complexes at appropriate metal ion-ligand-ratio is consistent with complexes observed with Schiff base and hydroxynaphthalene derivatives **1** and **2** with divalent metal ions [17, 33, 38]. Even though the data from the photometric titrations of **3** with Zn^2+^ and Pb^2+^ could not be used to determine the binding stoichiometry, complex structures may be assumed similar to those observed with Mg^2+^ and Fe^2+^. In analogy to the corresponding complexes of salicylaldehyde Schiff bases it is proposed that Zn^2+^ forms 2:1 complexes with **3** with an even higher binding affinity than the one with Mg^2+^ because the *d*-orbitals of Mg^2+^ do not interact significantly with the π electrons of the ligand resulting in a weaker binding [18]. However, the available data is inconclusive and does not allow to assign particular complex structures. Instead, the data just confirm that more than one absorbing entity in solution is formed by different development of separate bands during titration.

### Fluorimetric Detection of Metal Ions

**Fig. 2:**
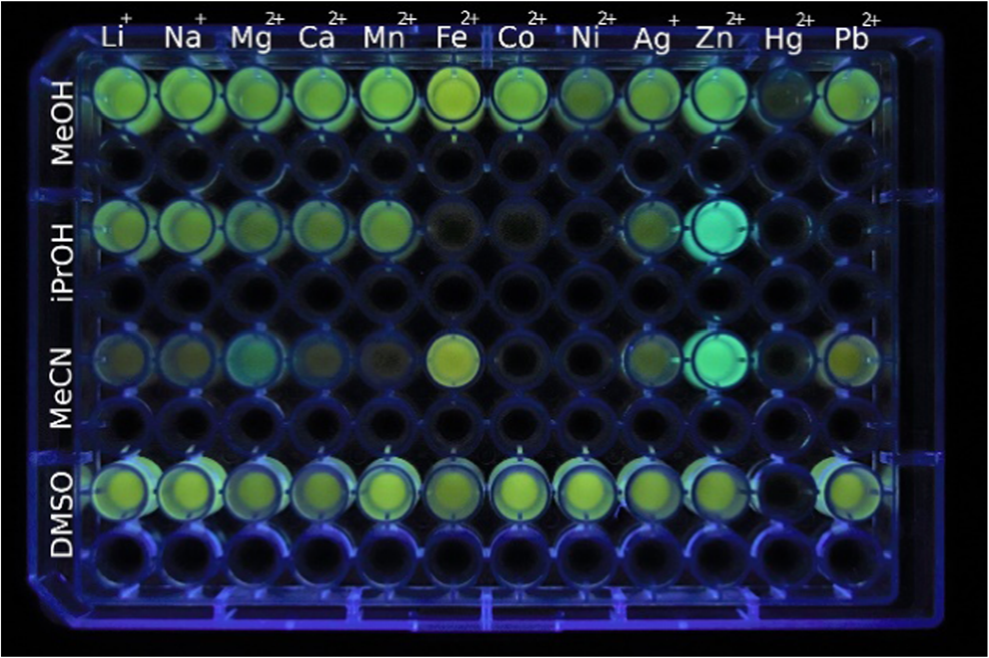
Emission of solutions containing **3** (*c* = 100 μM; *λ*_exc_ = 366 nm) in the presence of metal ions (*c* = 200 μM, lines 1, 3, 5, and 7) and blank samples (lines 2, 4, 6, and 8) in MeOH, 2-PrOH, MeCN, and DMSO

**Fig. 3:**
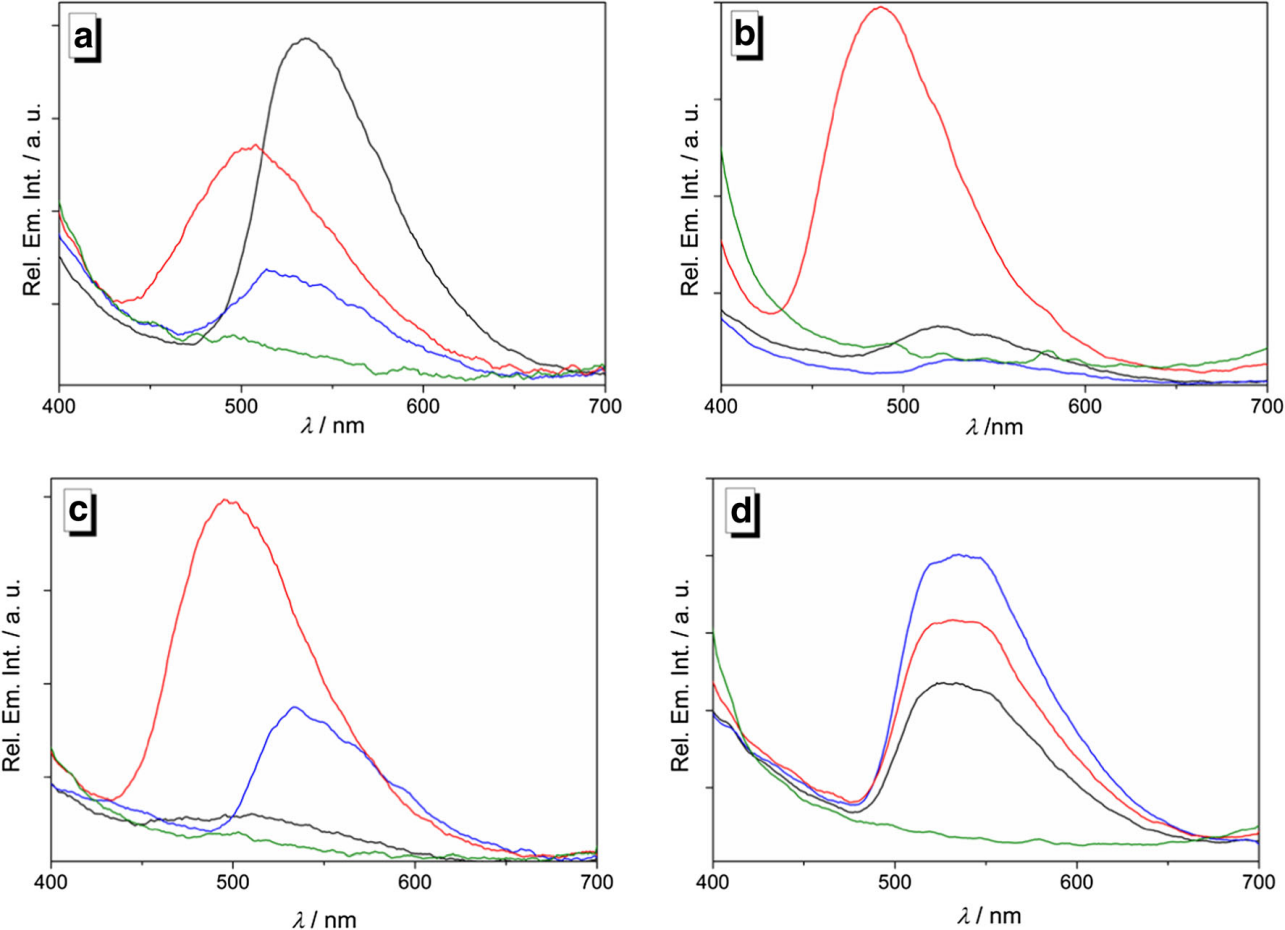
Emission spectra of **3** (*c* = 100 μM, *λ*_ex_ = 366 nm) in the presence of metal ions (*c* = 200 μM, black: Mg^2+^; red: Zn^2+^; blue: Fe^2+^; green: Hg^2+^) in (**a**) MeOH, (**b**) 2-PrOH, (**c**) MeCN and (**d**) DMSO

The changes of the emission properties of the isoquinoline **3** upon complexation of metal ions were also investigated. For that purpose, a series of representative metal ions was added to solutions of **3** in different solvents in a multi-well plate setup, and the changes of the emission color as well as the corresponding emission colors were determined (Figs. 2 and 3). Most notably, the shifts of the emission bands, and for that matter also the emission color, and the emission intensity revealed different behavior with varying solvent and metal ion. As a general trend, complexes of **3** with Hg^2+^ are essentially non-fluorescent in all tested solvents. At the same time, the solutions of the ligand-metal complexes showed broad bands with emission maxima at 517 nm for most metal ions in MeOH, with the most intense emission in the presence of Mg^2+^, whereas the emission of the solutions of **1** in the presence of Fe^2+^ and Zn^2+^ had emission maxima at 535 nm and 506 nm, respectively. In 2-PrOH, the addition of the alkali and earth alkali metal ions (Li^+^, Na^+^, Mg^+^, Ca^+^) or Mn^2+^ resulted in emission bands with maxima at 522 nm and 551 nm, whereas the addition of Zn^2+^ gave a blue-shifted emission maximum at 487 nm. No significant emission was observed in the presence of the remaining metal ions of the series (Co^2+^, Ni^2+^, Ag^+^, Hg^2+^, Pb^2+^).

In acetonitrile solution, only a few metal ion-ligand complexes gave a distinct emission. Namely, complexes with Mg^2+^ gave a weak emission with a maximum at 497 nm, and the complexes with Fe^2+^, Zn^2+^ and Pb^2+^ gave more intense emission bands at 538 nm, 496 nm and 530 nm. In contrast, solutions of **3** with Li^+^, Na^+^, Ca^2+^, Mn^2+^, Co^2+^, Ni^2+^ and Ag^2+^ had only a weak emission with a maximum at 530 nm.

In DMSO solution, a broad emission band around 530 nm was observed along with a blue-shifted band in the presence of nearly all metal ions of the series, except for Hg^2+^. Both of these bands match the ones of **3** in DMSO in the absence of metal ions, which may indicate that no significant binding occurs between the ligand and the tested metal ions in DMSO as a solvent.

In MeOH, 2-PrOH and acetonitrile, the emission observed in the presence of Zn^2+^ has the highest intensity or is amongst the highest in intensity within the group of tested cations, and the emission maximum is in all cases blue-shifted relative to the maxima observed with the other ions, except for Mg^2+^ in acetonitrile. Furthermore, solutions of the ligand **3** in 2-PrOH and acetonitrile that contained Co^2+^, Ni^2+^, Ag^+^ and Hg^2+^ showed little or no emission.

Notably, the diverse emission colors and intensities of the different complexes of compound **3** in various solvents can be used for a pattern analysis with which particular ions may be identified. Thus, the matrix shown in Fig. 2 with a combination of metal cation (abscissa) and solvent (ordinate) clearly shows characteristic patterns of emission color and intensity for Zn^2+^, Fe^2+^, Mg^2+^, and Pb^2+^, so that these ions are easily identified with compound **3** as fluorimetric probe by this method.

**Fig. 4:**
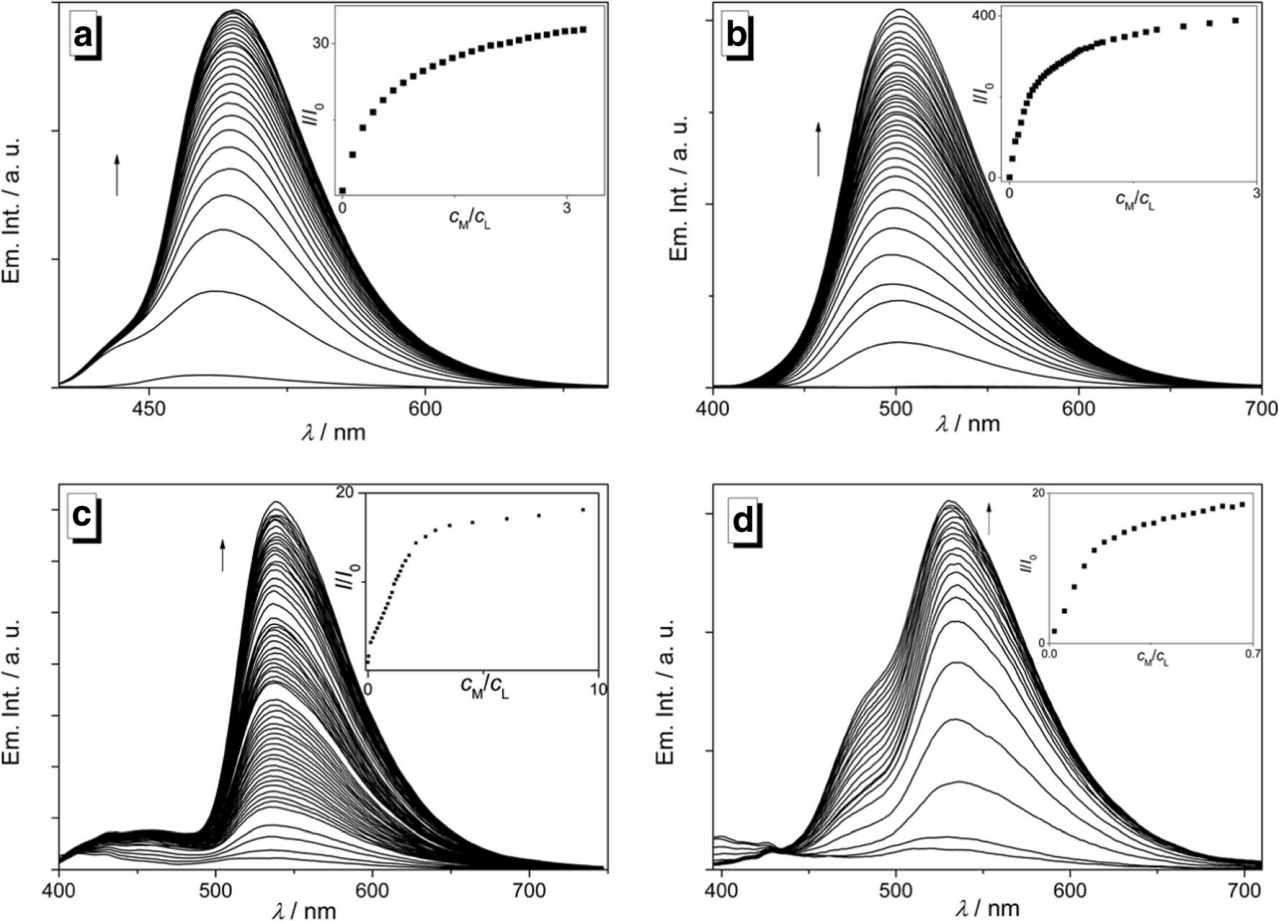
Fluorimetric titrations of **3** (*c*_*L*_ = 50.0 μM) with Mg^2+^ (**a**, *λ*_ex_ = 383 nm), Zn^2+^ (**b**, *λ*_ex_ = 380 nm), Fe^2+^ (**c**, *λ*_ex_ = 381 nm), and Pb^2+^ (**d**, *λ*_ex_ = 379 nm) in acetonitrile. The arrows indicate the development of emission bands during titration. Inset: Plot of the relative emission intensity *I* / *I*_0_ vs. *c*_M_/*c*_L_, *c*_M_ = conc. of the metal ion

Based on the preliminary screening results (Fig. 3), the emission properties of the hydroxypyridylisoquinoline **3** upon complexation with selected metal ions were investigated with fluorimetric titrations (Fig. 4). To this end, titrations of the metal complexes with Zn^2+^, Mg^2+^, Fe^2+^ and Pb^2+^ to ligand **3** were performed in acetonitrile solution, because in this solvent the differences between the emission properties of the resulting complexes as well as their emission intensities were most pronounced (Figs. 2 and 3). During the titration of **3** with Mg^2+^, Fe^2+^ and Pb^2+^, emission maxima developed at 497 nm, 538 nm and 530 nm, respectively. Conversely, the fluorimetric titration of **3** with Zn^2+^ gave an emission maximum at 496 nm with a fluorescence light-up effect that is 20 times stronger than the one observed with Mg^2+^, Fe^2+^ and Pb^2+^ (Fig. 4). The highest fluorescence quantum yields were obtained for complexes with Zn^2+^ (*Φ*_fl_ = 0.35) and Mg^2+^ (*Φ*_fl_ = 0.30), respectively, whereas the complexes with Fe^2+^ (*Φ*_fl_ = 0.05) and Pb^2+^ (*Φ*_fl_ = 0.01) exhibit significantly lower fluorescence quantum yields (Table 1). Notably, binding of **3** with Zn^2+^ gave a distinct single emission band while the complexation of Mg^2+^, Fe^2+^ and Pb^2+^ resulted in the formation of additional weak (Mg^2+^, Pb^2+^) or pronounced (Fe^2+^) blue-shifted bands or shoulders (Fig. 4, Table 1). It should be noted that the lower intensity in these higher-energy bands and shoulders is caused by the strong overlap with the lowest energy absorption bands, contributing to the low fluorescence quantum yields, especially in the case of solutions containing Fe^2+^ and Pb^2+^ where this inner filter effect is obviously particularly strong.

The complexation between the ligand **3** and the metal ions leads to different emitting complexes whose emission maximum and intensity depend on the employed metal ion and the solvent (Figs. 2 and 3). However, there is no obvious single parameter that seems to govern these emission properties, as indicated by missing relationships between emission intensity or energy and the solvent parameters, such as e.g. polarity, hydrogen bond donor or acceptor ability, dipole moment, dielectric constant, etc., or with the properties of the metal ion, such as size, ionization potential, redox potential, Lewis acidity, ligand sphere, etc. (Table S1) And even if only few binding constants were determined (Table 1) it appears that the complex stability does not directly correlate with its emission intensity. Moreover, the available absorption and emission data do not allow to deduce the particular deactivation pathways of the excited states that lead to the emission quenching observed in some cases.

In general, almost all complexes show a moderate and well detectable emission intensity in the polar protic and polar aprotic solvents MeOH and DMSO. And the emission bands do not vary strongly over the series of metal ions, with complexes of Zn^2+^ and Fe^2+^ showing the most pronounced deviations. As the shifts of the emission also do not differ strongly from the ones of uncomplexed **3** in MeOH (λ_em_ = 519 nm) or DMSO solution (λ_em_ = 529 nm) [39] it may be deduced that the emission of the complexes in MeOH originates mainly from the ligand with only marginal influence from the metal ion and that every efficient quenching by the metal cations is suppressed in this solvent, presumably by solvation of the cation or the complex. In contrast, the emission of particular complexes is significantly quenched in 2-PrOH and acetonitrile solutions, however, with completely different trends. Whereas in 2-PrOH similar emission is observed for most cations as in MeOH, the emission of **3** is quenched by complexation to Fe^2+^, Co^2+^, Ni^2+^, Hg^2+^, and Pb^2+^, which may be explained by the different solvating or coordinating properties of the sterically more demanding 2-PrOH molecules that enables inner-sphere or collision quenching by the then less shielded transition metals [42–45]. In acetonitrile solution, only complexes of **3** with Zn^2+^ and Fe^2+^, and to some extent with Mg^2+^ and Pb^2+^, exhibit significant detectable emission, whereas the emission of the other complexes is efficiently quenched. As acetonitrile is the solvent with the weakest complexation to cations in the series of applied solvents, as indicated by its low donor number [46], it may be concluded that only the complexes of **3** with Zn^2+^, Fe^2+^, Mg^2+^, and Pb^2+^ are sufficiently persistent in this solvent, as confirmed by the binding constants (Table 1), thus suppressing competing radiationless deactivation pathways.

**Fig. 5:**
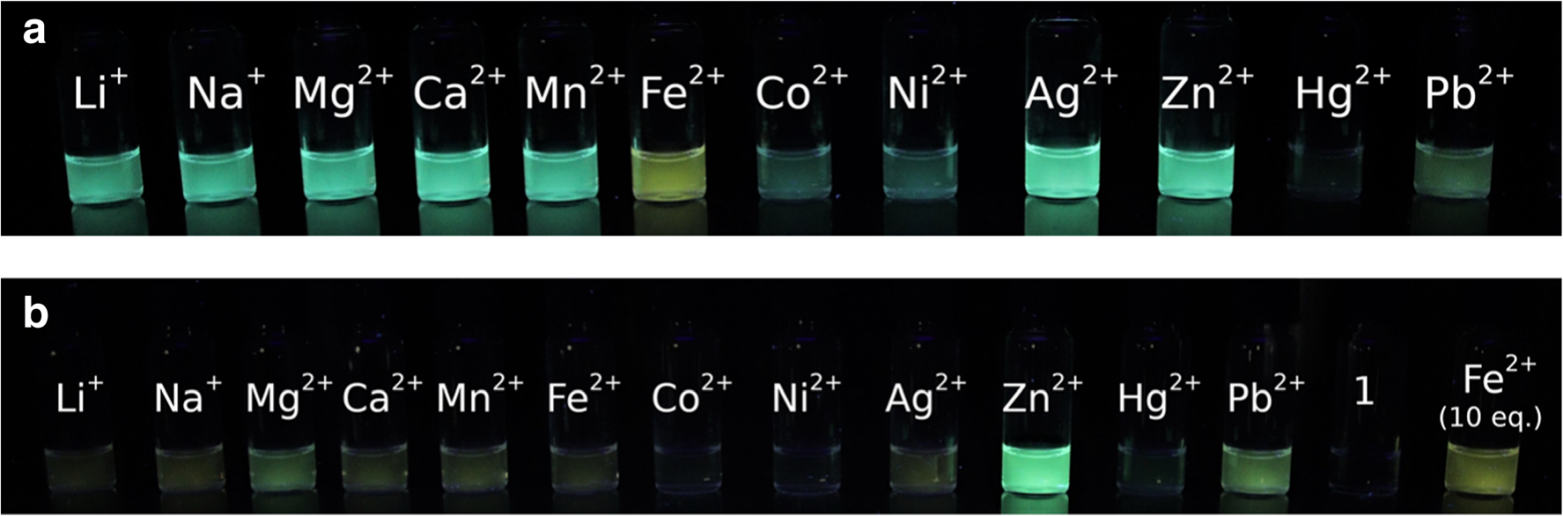
Emission of complexes of 3-hydroxy-4-pyridylisoquinoline (**3**) (*c* = 50 μM) and Zn^2+^ (**a**, *c*_M_ = 100 μM) or Fe^2+^ (**b**, *c*_M_ = 100 μM) in the presence of different metals (*c*_M_ = 1.00 mM) in acetonitrile solution

To assess the interference of other metal ions with the fluorimetric response to the complex formation of ligand **3** with Zn^2+^ and Fe^2+^, their emission color was determined in the presence of other, potentially competing metal ions (10 mol. Equiv.) in acetonitrile. Thus, the green emission of the complex between **3** and Zn^2+^ in acetonitrile remains essentially unchanged only in solutions when Li^+^, Na^+^, Mg^2+^, Ca^2+^, Mn^2+^ and Ag^2+^ are present, whereas in the presence of Fe^2+^ a yellow emission was observed. At the same time, quenching of the emission is observed in the presence of Co^2+^ and Ni^2+^ while larger quenching was observed along with a slight color change in the presence of Hg^2+^ and Pb^2+^ (Fig. 5). Similarly, the emission color of the complex between **3** and Fe^2+^ remains essentially the same only in Na^+^, Ca^2+^ and Ag^2 +^ containing solutions, whereas in the presence of an additional amount of Fe^2+^ a yellow fluorescence was observed assigned to the complex with this ion. A slight decrease in emission intensity was observed in the presence of Li^+^ while the emission is significantly quenched by Co^2+^, Ni^2+^, Hg^2+^ and Pb^2+^ (Fig. 5). These control experiments revealed that the presence of some particular cations interferes with the fluorimetric detection of Zn^2+^ and Fe^2+^ in acetonitrile solution, most likely because of competing complex formation or by collision-based quenching.

## Conclusion

In summary, we studied the interactions of 3-hydroxy-4-pyridylisoquinoline (**3**) with several metal ions and the corresponding effects on the absorption and emission properties. We demonstrated that the complexation of the metal ions leads to characteristic optical responses that depend significantly on the employed solvents, especially with Zn^2+^, Fe^2+^, and Mg^2+^. In case studies in acetonitrile solution, the complex formation with these selected cations by photometric and fluorimetric titrations revelealed some insights in the underlying equilibria and the origin of the detected emission. These effects cannot be used in general for the fluorimetric detection of cations in separate fluorimetric titrations with complex mixtures. But we have clearly demonstrated that the dependence of the emission intensity and energy of **3** on the nature of the cation and the solvent may be used for the fluorimetric identification of particular metal cations in a matrix-based pattern analysis, as shown in Fig. 2. Thus, the combined information from fluorimetric analyses in different solvents and comparison with the color pattern of other cations allows the unambiguous identification of Zn^2+^, Fe^2+^, Mg^2+^, and Pb^2+^, separately with one and the same probe. Moreover, the pronounced emission properties of the **3**-Zn^2+^ complex, specifically the exceptionally large light-up effect on complex formation, may be used to detect this cation fluorimetrically even from mixtures. Although the fluorimetric detection is not performed in aqueous solution as required for experiments under physiolocical conditions, at least the analysis of environmental samples is possible with this probe after appropriate preparation, i.e. lyophilization, and further analytical processing. From the overall results we conclude that the 3-hydroxy-4-pyridylisoquinoline (**3**) is a promising starting poing for the further development of cation-sensitive chemosensors. In particular, the parent compound and derivatives thereof [39] are readily accessible so that the selectivity of the metal ion complexation as well as the emission properties of the resulting complexes may be fine-tuned by the attachment of appropriate donor or acceptor substituents.

## Experimental Section

### Materials and Equipment

Absorption spectra were recorded with an Analytik Jena Specord S600 spectrophotometer in Hellma quartz cells 110-QS or 114B-QS (10 mm) with baseline correction at 20 °C. Emission spectra were collected with a Cary Eclipse spectrophotometer equipped with a microplate reader. Emission spectra were measured in Hellma quartz cells 114F-QS (10 mm × 4 mm) at 20 °C and in a UV-Star®, 96 well, μClear®, F-bottom well plate.

Compound **3** was synthesized according to published procedure [39]. All commercially available chemicals were reagent-grade and used without further purification unless otherwise mentioned. Spectroscopic grade solvents were used for solutions submitted to absorption and emission spectroscopy.

#### Methods

##### Spectrometric Titrations

Solutions were prepared for each experiment from stock solution of the metal ion and 3-hydroxy-4-pyridylisoquinoline (**3**) (*c* = 1.00 mM) in acetonitrile. Solutions for photometric and fluorimetric analysis were prepared by thoroughly evaporating appropriate amounts of stock solutions to complete dryness under a N_2_ stream and subsequent dissolution in MeOH, 2-PrOH, MeCN, or DMSO. The spectrometric titrations were performed according to published procedures [47]. The relative fluorescence quantum yields were determined according to standard protocol with Coumarin 153 (*Φ*_fl_ = 0.38 in EtOH) [41] as reference. The binding constants for ligand-metal ion complexes were determined with the *Bindfit* online tool for host-guest equilibria http://supramolecular.org [48, 49]. For the determination of the stoichiometry and binding constants of the complexes the binding isotherms at relevant wavelenghts were used in a fitting model based on the Nelder-Mead method contained in *Bindfit*, excluding any specification of cooperativity between the equilibria or any corrections from dilution http://supramolecular.org [48, 49].

##### Fluorimetric Detection of Metals Using a Well Plate

Appropriate amounts of stock solutions of **3** (*c* = 100 μM) and Li^+^, Na^+^, Mg^2+^, Ca^2+^, Mn^2+^, Fe^2+^, Co^2+^, Ni^2+^, Ag^2+^, Zn^2+^, Hg^2+^, or Pb^2+^ (*c* = 500 μM) were evaporated to dryness under a N_2_ stream in 96 bottom well plate and the residues were redissolved with MeOH, 2-PrOH, MeCN and DMSO. For this setup, odd rows contained solutions of **3** and metal ions, while the even rows were treated as blanks that contained solutions of metal ions. The emission spectra (*λ*_exc_ = 366 nm) were measured at 20 °C.

##### Assesment of Interference by Competing Metal Ions

Solutions containing 3-hydroxy-4-pyridylisoquinoline (**3**) (*c* = 50.0 μM), Zn^2+^ or Fe^2+^ (*c* = 100 μM) and a metal ion of the series (Li^+^, Na^+^, Mg^2+^, Ca^2+^, Mn^2+^, Fe^2+^, Co^2+^, Ni^2+^, Ag^2+^, Zn^2+^, Hg^2+^ or Pb^2+^; *c* = 1.00 mM) in acetonitrile were prepared by dilution of appropriate amounts of stock solution. The samples were irradiated with a UV lamp at *λ*_ex_ = 366 nm and photographed with a camera.

## Supplementary Information

ESM 1 (PDF 1146 kb)

## Data Availability

Not applicable.
