# Supplementary material for: Fluorimetric Detection of Zn2+, Mg2+, and Fe2+ with 3-Hydroxy-4-Pyridylisoquinoline as Fluorescent Probe
Source: J Fluoresc. 2020 Dec 19;31(1):269–77. doi: 10.1007/s10895-020-02666-0 (PMC7820078; doi:10.1007/s10895-020-02666-0)
Supplement: Supplementary file 1 — (PDF 1146 kb) [file 10895_2020_2666_MOESM1_ESM.pdf]

Supporting Information

Fluorimetric detection of Zn^{2+}, Mg^{2+}, and Fe^{2+} with 3-hydroxy-4-pyridylisoquinoline as fluorescent probe

Gabriel E. Gomez Pinheiro and Heiko Ihmels*

Department of Chemistry and Biology, and Center of Micro- and Nanochemistry and Engineering (*Cμ*), University of Siegen

Adolf-Reichwein-Str. 2, 57068 Siegen, Germany

Email: ihmels@chemie.uni-siegen.de

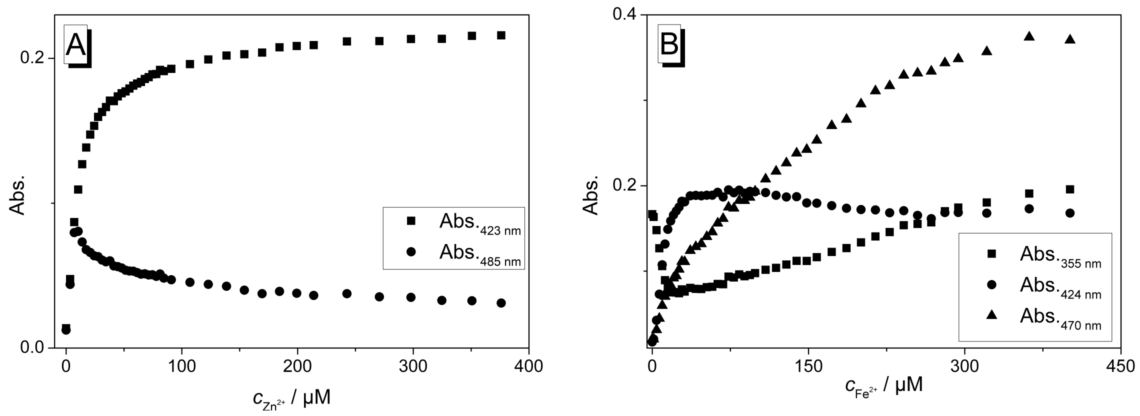

Figure S1. Plot of absorption versus metal ion concentration from photometric titration of **3** (c = 50.0 µM) with (A) Zn^{2+} and (B) Fe^{2+} in acetonitrile.

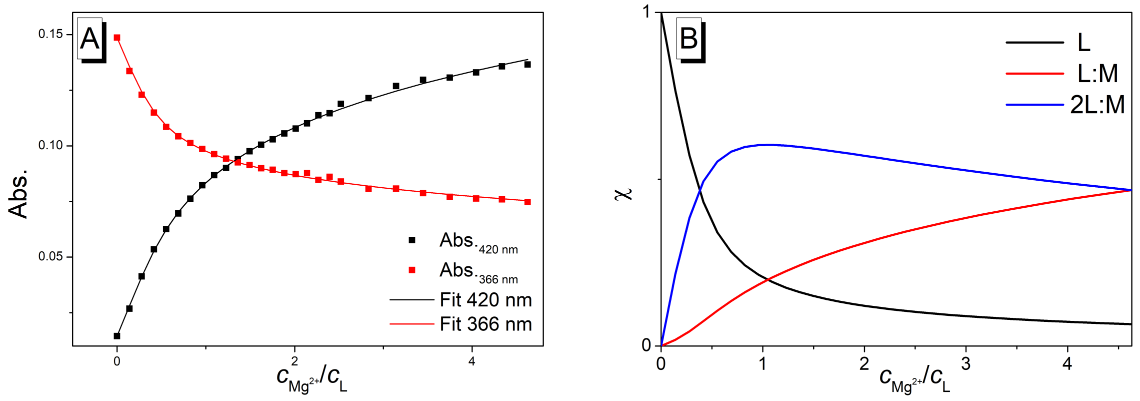

Figure S2. (A) Titration curves and (B) speciation diagram obtained from fitting of the photometric titration of **3** with Mg^{2+} in acetonitrile with *BindFit*.

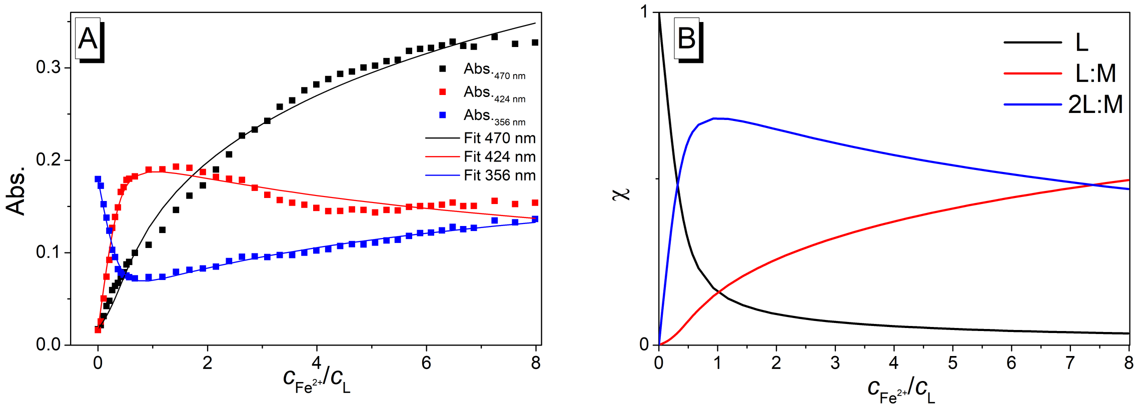

Figure S3. (A) Titration curves and (B) speciation diagram obtained from fitting of the photometric titration of **3** with Fe^{2+} in acetonitrile with *BindFit*.

Table S1. Emission maxima of **3** and its complexes with Mg^{2+}, Zn^{2+}, Fe^{2+} and Pb^{2+} in different solvents, along with the representative solvent parameters E_T^{30}, *α*, *β*, and *π**.

	$E_T^{30\ a}$ / kcal mol^{-1}	***α*** [a]	***β*** [a]	***π**** [a]	**3** [b]	**3** + Mg^{2+}	**3** + Zn^{2+}	**3** + Fe^{2+}	**3** + Pb^{2+}
					λ_{em} / nm				
MeOH	55.4	0.98	0.66	0.6	398, 519 [c]	542	506	535	535
2-PrOH	48.4	0.76	0.84	0.48	–	522, 551 [c]	487	527	–
CH_3CN	45.6	0.19	0.4	0.66	395	497	496	538	530
DMSO	45.1	0	0.76	1	411, 529 [c]	530	530	530	530

[a] E_T^{30}: solvent polarity; *α*: hydrogen bond donating ability, *β:* hydrogen bond accepting ability, *π**: dipolarity/polarizability polarity; (Ref. [1] and [2]. [b] Ref. [3]. [c] Dual emission.

References

[1] Reichardt C (1994) Solvatochromic Dyes as Solvent Polarity Indicators. Chem. Rev. 94: 2319–2358. https://doi.org/10.1021/cr00032a005

[2] Kamlet MJ, Abboud JLM, Abraham MH, Taft RW, (1983) Linear Solvation Energy Relationships. 23. A Comprehensive Collection of the Solvatochromic Parameters, *π**, *α*, and 0 *β*, and Some Methods for Simplifying the Generalized Solvatochromic Equation. J. Org. Chem. 48: 2877–2887. https://doi.org/10.1021/jo00165a018

[3] Gomez Pinheiro GE, Ihmels H, Dohmen C, (2019) Mild Synthesis of Fluorosolvatochromic and Acidochromic 3-Hydroxy-4-pyridylisoquinoline Derivatives from Easily Available Substrates. J. Org. Chem. 84: 3011–3016. https://doi.org/10.1021/acs.joc.8b03272
